# Pyrroloquinoline Quinone Alleviates Jejunal Mucosal Barrier Function Damage and Regulates Colonic Microbiota in Piglets Challenged With Enterotoxigenic *Escherichia coli*

**DOI:** 10.3389/fmicb.2020.01754

**Published:** 2020-07-24

**Authors:** Caiyun Huang, Dongxu Ming, Wenhui Wang, Zijie Wang, Yongfei Hu, Xi Ma, Fenglai Wang

**Affiliations:** ^1^State Key Laboratory of Animal Nutrition, College of Animal Science and Technology, China Agricultural University, Beijing, China; ^2^Department of Internal Medicine and Biochemistry, University of Texas Southwestern Medical Center, Dallas, TX, United States

**Keywords:** pyrroloquinoline quinone, antioxidant, inflammation, intestinal mucosa, tight junction, enterotoxigenic *Escherichia coli* K88

## Abstract

This study aimed to evaluate the effect of dietary supplementation with pyrroloquinoline quinone (PQQ) on gut inflammation and microbiota dysbiosis induced by enterotoxigenic *Escherichia coli* (ETEC). Twenty Duroc × Landrace × Yorkshire crossbred barrows were assigned to four groups: two *E. coli* K88 challenge groups and two non-challenge groups, each provided a basal diet supplemented with 0 or 3 mg/kg PQQ. On day 14, piglets were challenged with 10 mL 1 × 10^9^ CFU/mL of *E. coli* K88 or PBS for 48 h. The villus height (VH) and villus height/crypt depth (VCR) ratio of the *E. coli* K88-challenged group supplemented with PQQ was significantly reduced than in the non-supplemented challenge group (*P* < 0.05), while levels of jejunal zonula occludens-3 (ZO-3), diamine oxidase, secretory immunoglobulin A (SIgA), interleukin-10 (IL-10), and IL-22 proteins were higher (*P* < 0.05), as were the activities of glutathione peroxidase, total superoxide dismutase, and total antioxidant capability (*P* < 0.05). Moreover, PQQ supplementation alleviated an increase in levels of mucosal inflammatory cytokines and reduced the activity of nuclear factor-kappa B (NF-κB) pathway by *E. coli* K88 (*P* < 0.05). Gene sequencing of 16S rRNA showed dietary supplementation with PQQ in *E. coli* K88-challenged piglets attenuated a decrease in *Lactobacillus* count and butyrate, isobutyrate level, and an increase in *Ruminococcus* and *Intestinibacter* counts, all of which were observed in non-supplemented, challenge-group piglets. These results suggest that dietary supplementation with PQQ can effectively alleviate jejunal mucosal inflammatory injury by inhibiting NF-κB pathways and regulating the imbalance of colonic microbiota in piglets challenged with *E. coli* K88.

## Introduction

In addition to being an absorptive and digestive organ, the gut is also the body’s first line of defense against harmful substances that can cause intestinal inflammation or dysbacteriosis, both of which promote the development of intestinal diseases (Kiely et al., 2018; Mamantopoulos et al., 2018). Enterotoxigenic *Escherichia coli* (ETEC) invasion is a cause of intestinal damage and diarrhea in children and young animals (Gomes et al., 2016). Piglets are particularly prone to ETEC K88-induced diarrhea when the bacteria adhere to the small intestinal epithelium and produce adhesins and enterotoxins, ultimately causing intestinal inflammation via activation of the nuclear factor-kappa B (NF-κB) pathway (Chandler and Mynott, 1998; Yu et al., 2017). Invasion by ETEC K88 also disrupts the colonic microflora balance in piglets (Cheng et al., 2018). Antibiotics and heavy metal compounds like zinc oxide and copper sulfate are widely used in the swine industry to treat ETEC infections. However, serious concerns have arisen regarding the emergence of antibiotic-resistance bacteria and heavy metal pollution. Thus, it is vital to seek alternative ETEC treatments that positively affect animal gut health while producing fewer negative environmental side effects.

Pyrroloquinoline quinone (PQQ) is a water-soluble and heat-stable molecule (Duine et al., 1990) that plays an important role in several physiological and biological processes (Akagawa et al., 2016). Dietary supplementation with PQQ has been shown to improve neonatal growth and decrease redox potential in animals (Killgore et al., 1989; Wang et al., 2015). The weaned pups of female rats that were administered PQQ-supplemented diets during gestation and lactation could increase intestinal tight junctions proteins expression (Zhang et al., 2019). Furthermore, PQQ has been shown to regulate levels of jejunal inflammatory factors in weaned piglets (Zhang et al., 2020) and promote T-cell immunological memory during immune responses (Rucker et al., 2009). A previous study has also shown that PQQ supplementation prevents cecal microbial dysbiosis in the offspring of obese mice (Friedman et al., 2018). Moreover, we found in our previous study that dietary supplementation with PQQ improves intestinal development in weaned piglets (Yin et al., 2019). However, very little research that focuses on the influence of PQQ supplementation on ETEC K88-induced diarrhea in weaned piglets, and on the mechanisms by which PQQ might reduce intestinal damage, has been conducted.

We hypothesized that PQQ can alleviate intestinal injury by inhibiting activation of the NF-κB signaling pathway and regulating the balance of colonic microflora in weaned piglets. In this study, we used an ETEC K88-challenged piglet model to evaluate the effect of dietary supplementation with PQQ on the barrier function and inflammatory responses of the jejunum and colonic microflora.

## Materials and Methods

### Animals and Experimental Design

All experimental protocols of this study were approved by the Animal Care and Use Committee of China Agricultural University (AW03059102-1) and carried out in compliance with the National Research Council’s Guide for the Care and Use of Laboratory Animals. Twenty Duroc × Landrace × Yorkshire crossbred barrows were weaned on day 28 with initial body weight of 7.91 ± 0.192 kg. The piglets were randomly assigned to one of four treatment groups and were individually housed in metabolic cages (1.2 m × 0.4 m × 0.5 m) in well-ventilated, environment-controlled (29 ± 2°C) rooms. In order to avoid cross-contamination among pens during cleaning and feeding, implements exclusive to each pen were used during cleaning, which was carried out twice daily by an experienced worker. Each treatment comprised five replicates, with one piglet per replicate. The basal diet contained no antibiotics and provided sufficient nutrients to meet the National Research Council’s (NRC, 2012) nutrient requirements (Supplementary Table S1). All piglets had *ad libitum* access to feed and water throughout the study. The study consisted of four treatment groups: (1) piglets were offered the basal diet and not challenged with ETEC K88 (CTRL group); (2) piglets were offered the basal diet supplemented with 3 mg/kg PQQ⋅Na_2_ and not challenged with ETEC K88 (PQQ group); (3) piglets were offered the basal diet and challenged with ETEC K88 (CTRL + K88 group); and (4) piglets were offered the basal diet supplemented with 3 mg/kg PQQ⋅Na_2_ and challenged with ETEC K88 (PQQ + K88 group).

On day 14 of the experiment, piglets in the CTRL + K88 and PQQ + K88 groups were gavaged with 10 mL 1 × 10^9^ CFU/mL of ETEC K88 (serotype O149; China Institute of Veterinary Drug Control, Beijing, China), and piglets in the CTRL and PQQ groups were gavaged with 10 mL of PBS. Body temperature was recorded at 0, 4, 12, 24, and 48 h after ETEC K88 challenge, and piglets were euthanized for sampling at 48 h. The dose of ETEC K88 and the timeline to euthanize pigs were based on a previous study conducted in our lab (Huang et al., 2019).

### Sample Collection

After a 12-h overnight fast, blood samples were collected at 48 h after the ETEC K88 challenge. Serum was separated and stored at −20°C for further analysis. After humanely euthanizing all barrows for sampling, the mid-jejunal tissue (approximately 2 cm in length) was collected and stored in 4% paraformaldehyde solution for intestinal morphology analysis. A segment from the middle of the jejunum was cut open longitudinally and flushed with ice-cold PBS to collect mucosa. The middle portion of the colon was used to obtain luminal chyme, which was frozen rapidly in liquid nitrogen and stored at −80°C for further analysis.

### Dosage Information

Pyrroloquinoline quinone disodium (PQQ⋅Na_2_) (purity > 98%) was diluted with corn starch to a concentration of 1 g/kg mixture, and then blended proportionally into a premix before being added to the diet.

The basal diet was supplemented with PQQ⋅Na_2_ at a concentration of 3 mg/kg to provide an estimated 0.154 mg/kg body weight per day, based on average daily feed intake and average body weight (Supplementary Table S2). The molecular structure, dosages, and source of PQQ⋅Na_2_ are described in our previous study (Yin et al., 2019).

### Jejunal Morphology

The methods used for histological examination of the intestinal morphology have been described previously (Luna, 1968). Briefly, after 24 h of fixation with 4% paraformaldehyde solution, jejunal samples were dehydrated and embedded in paraffin. The embedded jejunal samples were cut to 5 μm sections and stained with hematoxylin and eosin. A microscope (Olympus BX-51; Olympus Optical Company) with imaging software (VisiVIEW, Visitron Systems GmbH) was used to evaluate villus height (VH), crypt depth (CD), and villus height/crypt depth ratio (VCR). For each sample, six slides were prepared with two separate sections per slide. At least 20 well-oriented villi and crypts were measured on each slide.

### Mucosal mRNA Expression

Total RNA was extracted from jejunal mucosa using HiPure Total RNA Mini Kit (R4114, Magen, Co., Guangzhou, China) and cDNA was synthesized using a reagent kit (RR037A, TaKaRa Bio, Inc., Japan) according to the manufacturer’s protocol. Quantitative real-time PCR was performed using the ABI 7500 Real-Time PCR system (Applied Biosystems, Foster City, CA, United States). The primers for target genes are listed in Supplementary Table S3. The relative mRNA expression of the target genes was normalized with expression of β-actin using by the 2^–ΔΔCT^ method (Pfaffl et al., 2002).

### Western Blotting

Frozen jejunal mucosa samples (100 g) were powdered in liquid nitrogen and lysed with RIPA buffer composed of protease and phosphatase inhibitors. The homogenate was centrifuged followed by sonication. A total of 80 μg protein in the supernatant from each sample was separated on 10% SDS polyacrylamide gels and transferred onto polyvinylidene fluoride membranes (IPVH00010, 0.45 μm; Merck & Co., Inc., Hunterton, NJ, United States), blocked with 5% skim milk in 1 × Tris–buffered saline, and incubated with the primary antibodies of myeloid differentiation factor 88 (MyD88, 23230-1-AP; ProteinTech, Chicago, IL, United States), inhibitor of nuclear factor-kappa B alpha (IκB, #4814; Cell Signaling Technology, Danvers, MA, United States), phosphorylated inhibitor of nuclear factor-kappa B alpha (p-IκB, #2859; Cell Signaling Technology), NF-κB (p65, #8242; Cell Signaling Technology), phosphorylated nuclear factor-kappa B (p-NF-κB [p65]; #3033, Cell Signaling Technology), interleukin-22 (IL-22, ab193813; Abcam, Cambridge, MA, United States), zonula occludens-3 (ZO-3, ab205882; Abcam), occludin (27260-1-AP; ProteinTech), and β-actin (#4970; Cell Signaling Technology). The samples were incubated overnight at 4°C, followed by further incubation with DyLight 800-conjugated secondary antibodies for 1 h at 25°C. The intensity of protein bands was determined with Odyssey Clx (Gene Company Limited, Hong Kong, China) and quantified using ImageJ software.

### Mucosal Factor Levels

The levels of IL-4, IL-6, IL-10, tumor necrosis factor α (TNF-α), and interferon gamma (INF-γ) were detected in the jejunal mucosa using commercial swine double-antibody sandwich enzyme-linked immunoassay (ELISA) kits (eBioscience, Inc., San Diego, CA, United States) according to the manufacturer’s instructions; absorbance was measured at 450 nm. The minimal detection limit was 1.3 pg/mL for IL-4, 0.92 pg/mL for IL-6, 1 pg/mL for IL-10, 5 pg/mL for TNF-α, and 4 pg/mL for INF-γ.

The level of secretory immunoglobulin A (SIgA) in the jejunal mucosa was detected via turbidimetric inhibition immunoassay, and diamine oxidase (DAO) activity was measured using the ELISA method. In order to detect the activities of glutathione peroxidase (GSH-Px), total superoxide dismutase (T-SOD), total antioxidant capability (T-AOC), malondialdehyde (MDA), and alkaline phosphatase (ALP), we used colorimetric methods according to product manuals of the corresponding commercial kits (Nanjing Jiancheng Bioengineering Institute, Nanjing, China). Absorbance was measured at 340 nm for SIgA, 460 nm for DAO, 412 nm for GSH-Px, 550 nm for T-SOD, 520 nm for T-AOC and ALP, and 532 nm for MDA. The minimal detection was 0.7 g/l for SIgA, 1.28 ng/mL for DAO, 20 U/mL for GSH-Px, 5 U/mL for T-SOD, 0.2 U/mL for T-AOC, 0.5 nmol/mL for MDA, and 3.07 U/l for ALP. The intra-assay CV was < 5% and inter-assay CV was < 6% for each assay.

### DNA Extraction and PCR Amplification

Microbial DNA from the colonic contents was extracted using a commercial kit (Omega Bio-Tek, Inc., Norcross, GA, United States) according to the manufacturer’s protocol. The final DNA concentration and purification were determined using the NanoDrop 2000 UV-vis spectrophotometer (Thermo Fisher Scientific, Wilmington, DE, United States) and DNA quality was assessed by 1% agarose gel electrophoresis. The V3–V4 hypervariable regions of the bacterial 16S rRNA gene were amplified with primers 338F (5’-ACTCCTACGGGAGGCAGCAG-3’) and 806R (5’-GGACTACHVGGGTWTCTAAT-3’) using a thermocycler (GeneAmp 9700; GeneAmp, ABI, United States). The PCR products were extracted using 2% agarose gel, and then purified using the AxyPrep DNA Gel Extraction Kit (Axygen Biosciences, Union City, CA, United States) and quantified using QuantiFluor^TM^-ST (Promega, United States) following the manufacturer’s protocol.

### Illumina MiSeq and Sequence Data Processing

Purified amplicons were pooled in equimolar concentrations and paired-end sequenced (2 × 300) on an Illumina MiSeq platform (Illumina, Inc., San Diego, CA, United States) according to the standard protocol by Majorbio Bio-Pharm Technology, Co., Ltd., (Shanghai, China). The raw reads were deposited into the NCBI Sequence Read Archive (SRA) database (Accession Number: SRP218537). Raw fastq files were quality and filtered, operational taxonomic units (OTUs) were clustered, and taxonomy of each 16S rRNA gene sequence was analyzed as previously described (Wu et al., 2019).

### SCFAs in Colonic Contents

The concentration of short-chain fatty acids (SCFAs) in colonic chyme was measured using a gas chromatographic method as previously described with slight modifications (Erwin et al., 1961). Briefly, 1.5 g of colonic content was suspended in 1.5 mL of distilled water and centrifuged at 15,000 × *g* for 10 min at 4°C. The supernatant (1 mL) was mixed with 200 μL of metaphosphoric acid in an ampoule, placed in an ice bath for 30 min, and then centrifuged for 10 min. An HP 19091N-213 column (30 m × 0.32 mm; Agilent, Santa Clara, CA, United States) was used in the HP 6890 Series Gas Chromatograph (Hewlett Packard, Paolo Alto, CA, United States). The injector and detector temperatures were maintained at 185 and 210°C, respectively. Each sample was measured three times.

### Statistical Analysis

Data were evaluated with one-way ANOVA using the Student-Newman–Keuls test. Values are expressed as mean ± SEM. Figures were created using GraphPad Prism 6. Bacterial diversity with standardized OTU reads was analyzed using R software. The bacterial community in the colonic digesta was analyzed using the Kruskal–Wallis H test; bacterial abundance at the order and genus levels are presented in bar plots. Linear discriminant analysis (LDA) effect size (LEfSe) analysis with an LDA threshold of > 2 was used to quantify biomarkers in each group before using the unpaired Wilcoxon rank-sum test to identify the most diverse group. Differences were regarded as significant at *P* < 0.05.

## Results

### Rectal Temperature, Intestinal Morphology, and Mucosal Barrier Function

**FIGURE 1:**
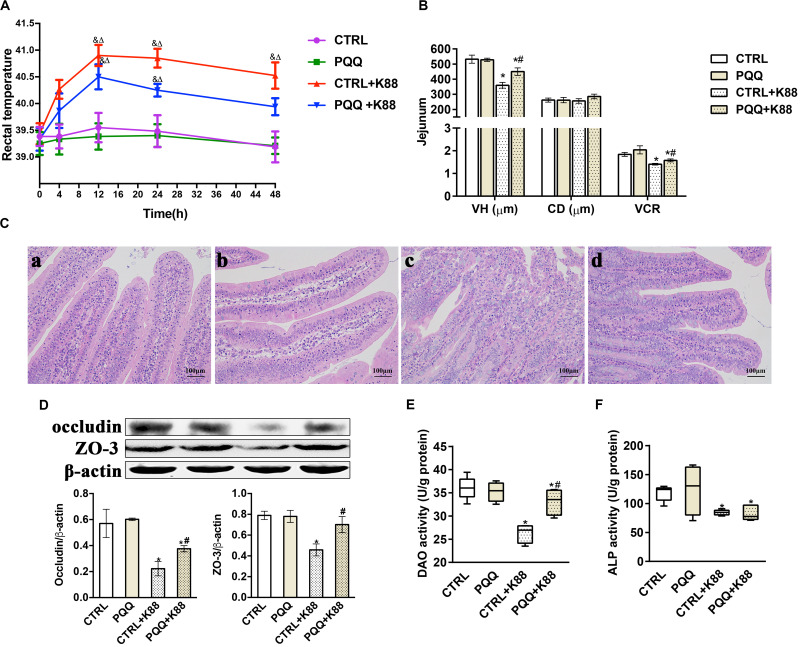
Effect of pyrroloquinoline quinone (PQQ) on rectal temperature and jejunal morphology in piglets challenged with enterotoxigenic *Escherichia coli* (ETEC) K88. **(A)** Body temperature of piglets was measured using digital thermometers; peak temperature was measured 12 h after the challenge. **(B)** The villus height (VH), crypt depth (CD), and villus height/crypt depth ratio (VCR) of the jejunum. **(C)** Hematoxylin-eosin staining was used to observe the jejunal morphology. **(C-a)** is from the group fed the basal diet (CTRL group); **(C-b)** is from the group fed the basal diet with PQQ and not challenged with ETEC K88 (PQQ group); **(C-c)** is from the group fed the basal diet without PQQ and challenged with ETEC K88 (CTRL + K88); and **(C-d)** is of the group fed the basal diet supplemented with PQQ and challenged with ETEC K88 (PQQ + K88). **(D)** Western blot analysis of occludin and ZO-3, and the densitometric values of tight junctions normalized to β-actin expression. **(E,F)** Protein levels of DAO and ALP in the jejunum. CTRL, piglets fed the basal diet; PQQ, piglets fed the basal diet supplemented with PQQ; CTRL + K88, piglets fed the basal diet and challenged with ETEC K88; PQQ + K88, piglets fed the basal diet supplemented with PQQ and challenged with ETEC K88. & represents comparison of hourly body temperature with that recorded 0 h after treatment (*P* < 0.05). δ represents comparison of hourly body temperature with the temperature of the CTRL group at the corresponding hour (*P* < 0.05). * denotes a significant difference (*P* < 0.05) from the CTRL group. # denotes a significant difference (*P* < 0.05) from the CTRL + K88 group. Data are presented as mean ± SEM; *n* = 5.

Changes in the rectal temperature of pigs in the PQQ group were similar to those in the CTRL group during the 48-h period after the challenge (Figure 1A). The rectal temperature in both groups gradually increased for 12 h, and then declined. Notably, the rectal temperatures in the PQQ + K88 group were significantly reduced at 48 h compared with those in the CTRL + K88 group. The VH and VCR of the jejunum were significantly reduced (*P* < 0.05) in the CTRL + K88 group compared with those in the other three groups (Figure 1B). Jejunal villi showed a clear structure in the CTRL and PQQ groups (Figures 1C-a,-b), and the border of the central lacteal was intact and exhibited very few infiltrative inflammatory cells. In the CTRL + K88 group (Figure 1C-c), the structure of jejunal villi was non-distinct, and the border of the central lacteal was less well-defined, with extensive inflammatory cell infiltration and lymph cell proliferation. Some lymph cell proliferation and inflammatory cell infiltration were observed in the PQQ + K88 group; however, the integrity of the villi and central lacteal were maintained (Figure 1C-d). The jejunal mucosal occludin and ZO-3 levels (Figure 1D), and DAO (Figure 1E), and alkaline phosphatase (ALP, Figure 1F) activities were reduced in the CTRL + K88 group (*P* < 0.05) relative to those in the PQQ + K88 group, demonstrating that the adverse effects on jejunal integrity from the K88 challenge were alleviated to a certain extent (*P* < 0.05) by PQQ supplementation.

### Protein and mRNA Abundance of Mucosal Cytokines in Jejunum

**FIGURE 2:**
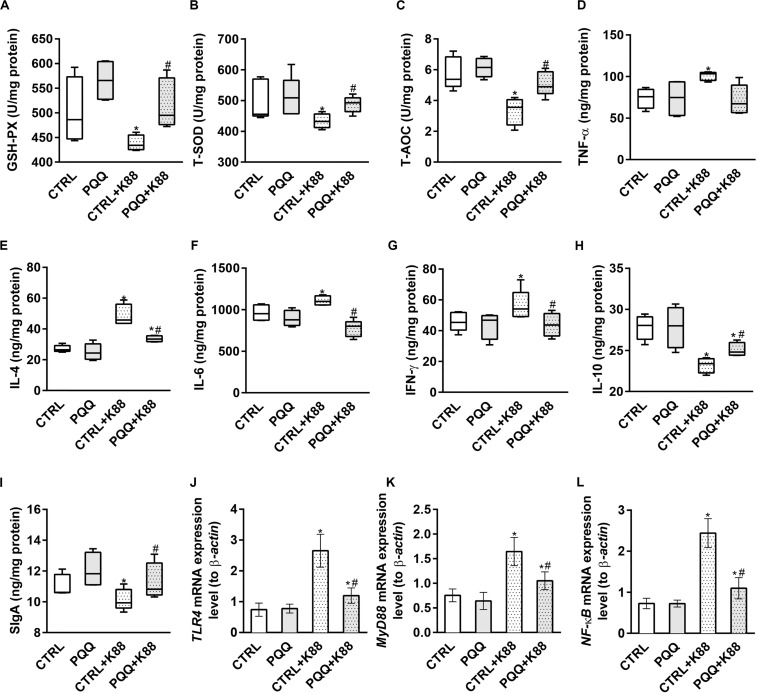
Effects of PQQ supplementation on mucosal cytokine expression in the jejunum after ETEC K88 challenge in weaned pigs. **(A–C)** The activities of antioxidant enzymes in the mucosa. **(D–I)** The levels of immune and inflammatory cytokines in the jejunum. **(J–L)** Data were obtained by real-time PCR; β-actin was the housekeeping gene. CTRL, piglets fed the basal diet; PQQ, piglets fed the basal diet supplemented with PQQ; CTRL + K88, piglets fed the basal diet and challenged with ETEC K88; PQQ + K88, piglets fed the basal diet supplemented with PQQ and challenged with ETEC K88. * denotes a significant difference (*P* < 0.05) from the CTRL group. # denotes a significant difference (*P* < 0.05) from the CTRL + K88 group; *n* = 5.

**FIGURE 3:**
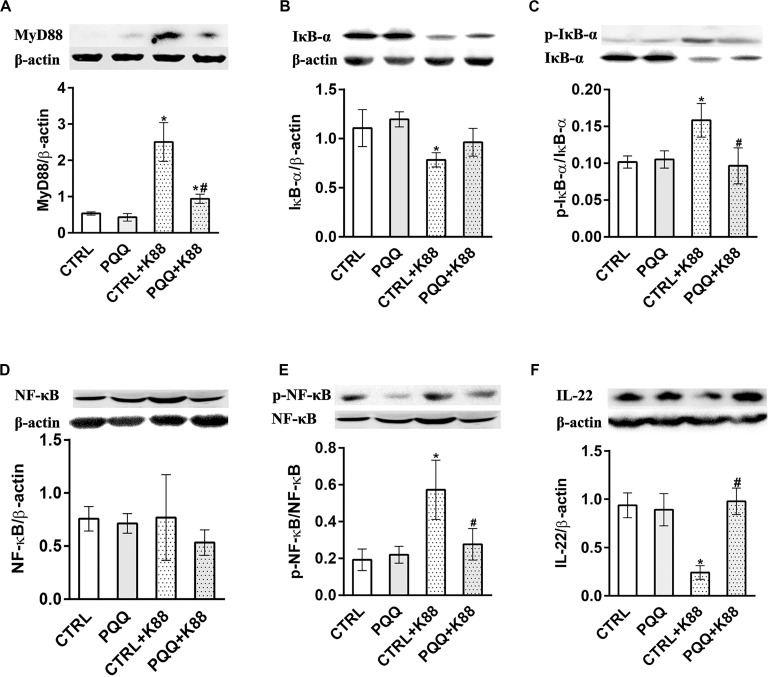
Effects of PQQ supplementation on the expression of proteins within the NF-κB signaling pathway in the jejunum after ETEC K88 challenge in weaned piglets. **(A–F)** Expression levels of MyD88, IκB-α, p-IκB-α, NF-κB, p-NF-κB, and IL-22 proteins are shown. CTRL, piglets fed the basal diet; PQQ, piglets fed the basal diet supplemented with PQQ; CTRL + K88, piglets fed the basal diet and challenged with ETEC K88; PQQ + K88, piglets fed the basal diet supplemented with PQQ and challenged with ETEC K88. * denotes a significant difference (*P* < 0.05) from the CTRL group. # denotes a significant difference (*P* < 0.05) from the CTRL + K88 group; *n* = 5.

Decreased activities of GSH-Px, T-SOD, and T-AOC (*P* < 0.05, Figures 2A–C) and increased levels of MDA (*P* < 0.05, Supplementary Figure S1A) were observed in the CTRL + K88 group compared with the CTRL group. Interestingly, the stimulatory effects of ETEC K88 on antioxidant indexes were reversed in the PQQ + K88 group. At 48 h, we observed an increase in the protein and mRNA levels of IL-4, IL-6, INF-γ, and TNF-α, and the mRNA level of IL-17 (*P* < 0.05; Figures 2D–G and Supplementary Figures S1B–F), along with a decrease in the protein expression of IL-10, SIgA (Figures 2H,I), and IL-22 (Figure 3F; *P* < 0.05) in the CTRL + K88 group compared with the CTRL group. The elevation in the level of pro-inflammatory cytokines and the expressions of SIgA, IL-10, and IL-22 were significantly attenuated in the PQQ + K88 group (*P* < 0.05).

### Activation of the NF-κB Pathway in the Jejunum

The jejunal mucosa of piglets in the CTRL + K88 group showed a significant (*P* < 0.05) increase in the mRNA expression of TLR4, MyD88, and NF-κB (Figures 2J–L) and the abundance of MyD88, IκB-α, p-IκB-α, and p-NF-κB (Figures 3A–C,E) compared with that of the CTRL group. However, this was not the case for NF-κB (*P* > 0.05, Figure 3D). The increased concentration of TLR4, MyD88, p-IκB-α, and p-NF-κB (*P* < 0.05) were attenuated in the PQQ + K88 group compared with those in the CTRL + K88 group.

### Colonic Microbiota Composition

**FIGURE 4:**
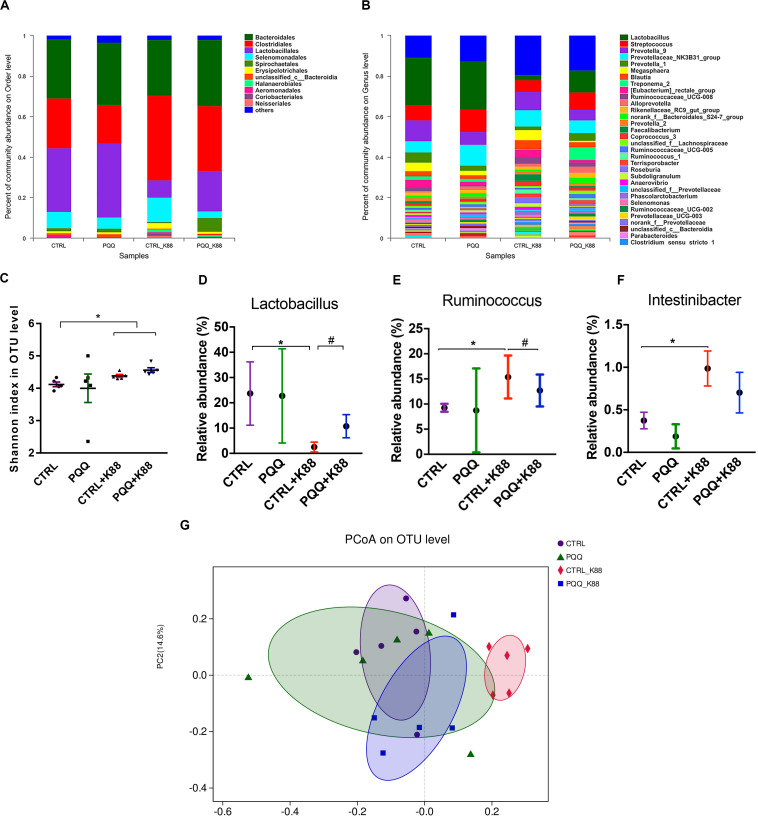
Colonic microbiota composition after PQQ supplementation in ETEC K88-challenged piglets. Relative contribution of order **(A)** and genus **(B)** in the colon of piglets in the CTRL, PQQ, CTRL + K88, and PQQ + K88 groups. **(C)** The α diversity, including Shannon curves, shows the diversity and richness of colonic microbiota in all groups. **(D–F)** The relative abundances of selected bacterial genera in the four groups are shown. **(G)** The dispersion of each sample in the four groups is reflected by the β diversity, PCoA score plot, and Bray–Curtis score plot. The abundance of taxa in the community is indicated by the diameter of each circle. Values are presented as mean ± SEM; *n* = 5. * denotes a significant difference (*P* < 0.05) compared with the control group. ^#^ denotes a significant difference (*P* < 0.05) compared with the CTRL + K88 group.

We further studied the effects of PQQ supplementation on colonic microbiota composition under inflammatory conditions. A Venn diagram of colonic samples from piglets in the CTRL, PQQ, CTRL + K88, and PQQ + K88 groups showed 750, 718, 744, and 718 OTUs, respectively. Among these, we found 622 common bacteria and 31 unique to specific groups (Supplementary Figure S2A). The relative abundance of colonic microbiota was determined at the phylum, order, and genus levels. Firmicutes, Bacteroidetes, Spirochetes, Proteobacteria, and Actinobacteria accounted for more than 90% of the total colonic bacterial community at the phylum level (Supplementary Figure S2B). At the order level, Bacteroidales, Clostridiales, Lactobacillales, Selenomonadales, and Spirochaetales were predominant. The combined abundance of Clostridiales and Selenomonadales was 24.5 and 41.6% in the CTRL and CTRL + K88 groups, respectively, whereas that of Lactobacillales was 31.5 and 8.4%, respectively; this level was restored in PQQ-supplemented, K88-challenged piglets (Figure 4A). *Lactobacillus, Streptococcus*, and *Prevotella* were the major genera found in the colonic contents of piglets (Figure 4B). The Shannon index of the CTRL + K88 and PQQ + K88 groups were higher than that of the CTRL group (*P* < 0.05, Figure 4C); however, this was not the case for the Chao1 index (*P* > 0.05, Supplementary Figure S2C). The abundance of *Lactobacillus* was lower in the CTRL + K88 group than in the CTRL group (*P* < 0.05), whereas that of *Ruminococcus* and *Intestinibacter* were more abundant in the CTRL + K88 group than in the CTRL group. The richness of these bacterial genera was restored in the PQQ + K88 group to the levels found in the CTRL group (Figures 4D–F). The β-diversity analysis by principal coordinates analysis (PCOA; Figure 4G) revealed a significant separation of clusters between the CTRL + K88 and PQQ + K88 groups. The predominant bacteria and maximum differences among taxonomic categories were determined across all four test groups using the LEfSe method, and are shown in a cladogram (Supplementary Figure S2D). Based on the cladogram, the following bacterial taxa were found significantly enriched in the PQQ + K88 group compared with those in the K88 group: Clostridia and Negativicutes classes; Clostridiales, Selenomonadales, and Pseudomonadales order; Lachnospiraceae, Ruminococcaceae, Peptostreptococcaceae, Veillonellaceae, and Moraxellaceae families; *Faecalibacterium, Butyricicoccus*, *Ruminococcus*, *Lachnospiraceae*_ND3007_group, *Intestinibacter*, and *Turicibacter* genera.

### Concentration of Colonic SCFAs

**FIGURE 5:**
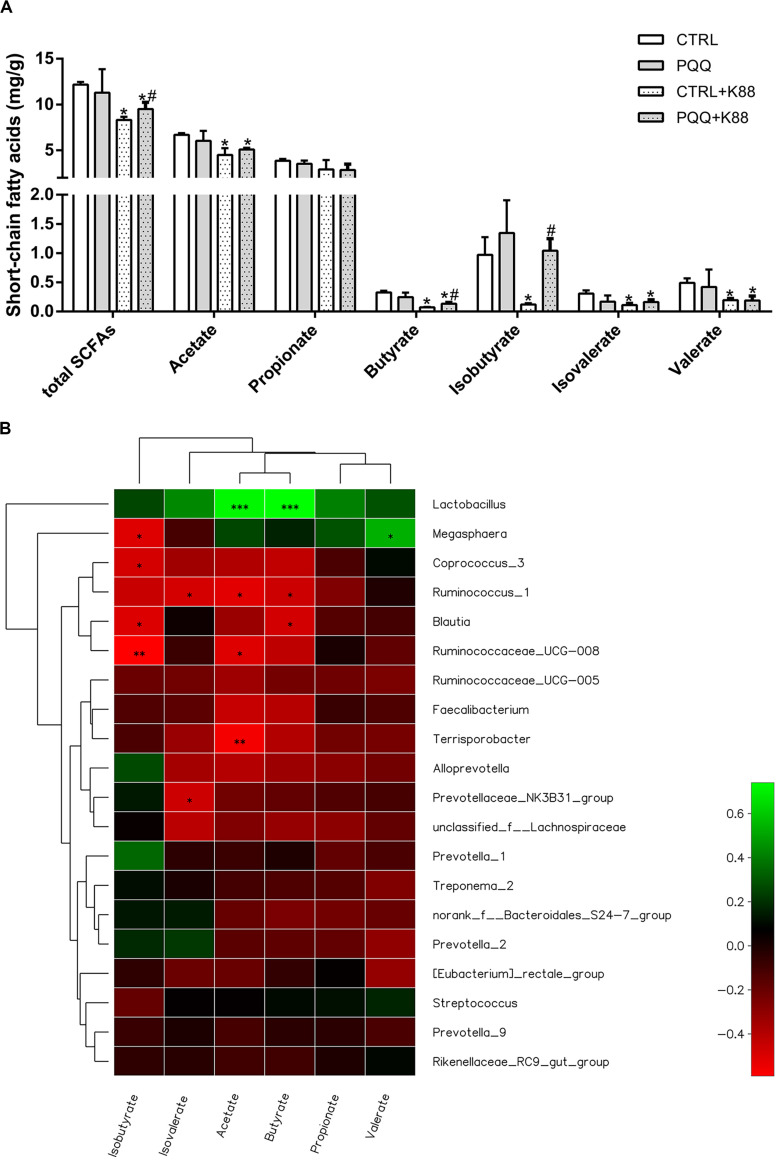
Effects of dietary PQQ supplementation on colonic short-chain fatty acids (SCFAs) in weaned piglets and correlation heatmap of the 20 most abundant genera and SCFAs. **(A)** Concentration of SCFAs in the chyme of the colon. Data are shown as mean ± SEM. The result was analyzed using the one-way ANOVA with Tukey’s test. ^∗^denotes a significant difference (*P* < 0.05) compared with the CTRL group. # denotes a significant difference (*P* < 0.05) compared with the CTRL + K88 group. **(B)** *X* and *Y* axes present SCFAs and genus, respectively. The correlation coefficients (R) are shown in different colors on the right side of the legend. The value of 0.01 < *P* ≤ 0.05 is marked with “^∗^.” The value of 0.001 < *P* ≤ 0.01 is marked with “^∗∗^.” The value of *P* ≤ 0.001 is marked with “^∗∗∗^”; *n* = 5.

Concentrations of total SCFAs, acetate, butyrate, isobutyrate, isovalerate, and valerate were lower in the CTRL + K88 group than in the CTRL group (*P* < 0.05), whereas those of total SCFAs, butyrate, and isobutyrate were significantly higher in the PQQ + K88 group than in the CTRL + K88 group (*P* < 0.05; Figure 5A). The correlation heatmap shows the relationship between bacterial genus and SCFAs. *Lactobacillus* showed a highly positive correlation with acetate and butyrate; *Megasphaera* presented a positive correlation with valerate and a negative correlation with isobutyrate; *Ruminococcus_*1 demonstrated a negative correlation with isovalerate, acetate, and butyrate; *Blautia* was negatively correlated with isobutyrate and butyrate; *Ruminococcacrae*_UCG-008 showed a negative correlation with isobutyrate and acetate; *Terrisporobacter* presented a highly negative correlation with acetate; and *Prevotellaceae*_NK3B31 demonstrated a significant negative correlation with valerate (*P* < 0.05, Figure 5B).

## Discussion

Diarrheal illness induced by ETEC is regarded as a severe public health concern and is a primary factor of morbidity and mortality among animals (Patterson et al., 2008). The growth-promoting agent PQQ (Samuel et al., 2015) has been shown to have antioxidant (Huang et al., 2017), anti-inflammatory, and immune-regulative functions (Jonscher et al., 2017). A recent study shows that dietary PQQ supplementation inhibits jejunal inflammation in piglets on day 28 after weaning (Zhang et al., 2020). In our previous study, we showed that dietary supplementation with 3 mg/kg PQQ⋅Na_2_ improved intestinal morphology, especially in the jejunum, and immunity in piglets (Yin et al., 2019). However, the role of PQQ in protecting the intestinal health of piglets against invasion from enteropathogenic bacteria is still unclear. Here, we used an ETEC K88-challenged piglet model to determine the effects of PQQ supplementation in modulating intestinal tissue injury and microbial composition.

Infection by ETEC K88 impairs intestinal integrity, which primarily manifests as damage to intestinal morphology and compromised barrier function, as reflected by VH, CD, and VCR (Liu et al., 2008). In the present study, we observed that the ETEC K88 challenge decreased VH and VCR in piglets; this is consistent with the findings of a previous study (Xun et al., 2015). An important marker of intestinal barrier function is DAO, which is abundant in enterocytes at the tips of small intestinal villi and is released into circulation if intestinal cells are injured (Wofoekamp and De Bruin, 1994). In our study, the jejunal DAO activity decreased after the ETEC K88 challenge. This result is similar to that observed in a study examining piglets after an *E. coli* lipopolysaccharide challenge (Wang et al., 2017). Moreover, ZO-3 and occludin, as key components of tight junctions, play key roles in the structure and permeability of the intestinal epithelia (Tsukita et al., 2001). In this study, the expression of jejunal ZO-3 and occludin proteins significantly decreased following ETEC K88 infection. These results demonstrate intestinal barrier injury after the ETEC K88 challenge.

Inflammatory reactions in subjects following an ETEC challenge are characterized by elevated rectal temperature (Lee et al., 2017). In our study, piglets showed fever, shivering, and inactivity within 4 h of the challenge, and diarrhea after approximately 1 day. Previous studies have reported that inflammatory cytokines in epithelial cells, such as TNF-α and IFN-γ, can impair tight-junction function, resulting in structural changes of the intestine (Roselli et al., 2003). In the present study, inflammatory cytokine expression increased in piglets challenged with ETEC K88. Che et al. (2017) similarly reported induction of an inflammatory response in the jejunum of pigs following ETEC K88 infection. Inflammatory cytokines are mainly associated with the activation of the NF-κB signaling pathway. The toll-like receptor (TLR) family consists of transmembrane receptors that play a role in recognizing pathogen-associated molecular patterns (Li and Verma, 2002). Within this family, TLR4 is a receptor of endotoxins from gram-negative bacteria such as ETEC K88, which transmits a signal to MyD88, causing phosphorylation of IκB-α. This, in turn, induces IκB degradation and NF-κB phosphorylation; the latter is then released into the nucleus, where it induces the expression of inflammatory cytokines (Velloso et al., 2015). The results of the present study showed that the levels of p-NF-κB and p-IκB-α increased after the ETEC K88 challenge. This indicates that ETEC K88 activates NF-κB pathway signaling and leads to an increase in inflammatory cytokines, which ultimately elicits a decline in the level of tight junction proteins and a loss of jejunal mucosal integrity.

Previous studies have shown that altered diversity of intestinal microbiota is a common phenomenon in several intestinal inflammation disorders, such as Crohn’s disease and irritable bowel syndrome (Frank et al., 2007). As a measure of diversity, we used the Shannon index (Willing et al., 2011) to assess microbial community diversity. We observed that ETEC K88 infection increased the Shannon index in piglets.

Although the infection of ETEC K88 did not significantly affect the composition of gut microbiota at the phylum level, we observed a considerable enrichment of the order Clostridiales under ETEC K88 challenge conditions. A similar study reported that gut inflammation in mice is associated with an increase in the relative proportion of Clostridiales (De La Serre et al., 2010). The genera of *Ruminococcus* and *Intestinibacter*, which belong to Clostridiales, were more abundant in the ETEC K88 group than in the other three treatment groups. Previous studies have consistently reported that the abundance of *Ruminococcus* and *Intestinibacter* are associated with intestinal inflammation (Berry et al., 2012). We also found a decreasing trend in the abundance of Lactobacillales in piglets challenged with ETEC K88. *Lactobacillus*, one of the richest genera of the order Lactobacillales, has a protective effect against atrophy via various mechanisms, including alteration of the gastrointestinal tract (Ozdemir, 2010). An abundant *Lactobacillus* population plays an important role in inhibiting the production of pathogenic bacteria like *E. coli*, as well as in recovery from intestinal injury (Wang et al., 2019). Notably, SCFAs are the byproducts of microbiotic fermentation, and mainly comprise acetate, propionate, and butyrate. These acids have been shown to inhibit stimulus-induced production of adhesion molecules and chemokines, and to exert anti-inflammatory effects (Xun et al., 2015). In the present study, we found decreased levels of SCFAs in ETEC K88-challenged piglets. The results described above demonstrate that we successfully established an ETEC K88 infection model in piglets, characterized by jejunal mucosal inflammation and colonic microbiota dysbiosis, and consequently intestinal barrier dysfunction.

We observed that the examined parameters (rectal temperature, VH, and occludin and ZO-3 expression in the jejunum) were effectively restored after the ETEC K88 challenge in piglets that had been treated with dietary PQQ supplementation. Therefore, we conclude that PQQ supplementation attenuates intestinal injury by regulating intestinal inflammatory responses. The present findings showed that the levels of SIgA and anti-inflammatory cytokines such as IL-22 and IL-10 increased in PQQ-supplemented piglets challenged with ETEC K88. All these factors play an important role in inhibiting the inflammation response. SIgA is the first line of defense against pathogenic bacteria and enteric toxins; it is secreted by the mucosa-associated lymphoid tissue (Boyaka, 2017). IL-10 is a pleiotropic cytokine that averts tissue damage by preventing excessive pro-inflammatory cytokine secretion, and it is secreted by Th2 and T-regulatory type 1 cells. IL-22 is a cytokine belonging to the IL-10 family, which can modulate the integrity of organs and tissues (Lindemans et al., 2015).

Our results show that PQQ supplementation before an ETEC K88 challenge in piglets can alleviate ETEC K88-induced oxidative stress and inflammatory reactions by upregulating antioxidant enzyme activities (GSH-Px, SOD, and T-AOC), and by reducing the mRNA expression of pro-inflammatory cytokines. Normally, excessive reactive oxygen species (ROS) in cells can cause oxidative stress to trigger excess production of pro-inflammatory factors and chemokines, resulting in inflammation. The main enzymes of the antioxidant system that scavenge ROS are GSH-Px, SOD, and chloramphenicol acetyltransferase (Tanida et al., 2011). Oxidative stress has been reported to activate NF-κB, and to ultimately promote NF-κB translocation from the cytoplasm to the nucleus (Pérez et al., 2017). In a previous study, dietary supplementation of PQQ decreased the activity of NF-κB in the jejunum of piglets 28 days after weaning (Zhang et al., 2020). Our current findings show that PQQ supplementation significantly decreases the abundances of p-NF-κB and p-IκB-α proteins in the jejunum of ETEC K88-challenged piglets. Therefore, we have demonstrated that PQQ can attenuate the oxidative stress and inflammatory response induced by ETEC K88 infection in the intestines of piglets by inhibiting the NF-κB pathway.

Our results indicate that the PQQ diet reversed ETEC K88-induced colonic microbiota dysbiosis by increasing the *Lactobacillus* count and levels of butyrate, isobutyrate, and total SCFAs, and by decreasing the abundance of *Ruminococcus* in the colon. The Spearman correlation heatmap shows a highly significant positive correlation among *Lactobacillus* count, acetate level, and butyrate level. These results indicate that PQQ may regulate a microbiotic shift toward higher *Lactobacillus* abundance, which may induce the production of SCFAs, such as butyrate, that favor gut health and regeneration (Tonel et al., 2010). Although we examined colonic microbiota, we were unable to study the effect of PQQ on jejunal microbial communities. This is because the 12-h fast before euthanasia depleted the level of chyme in the foregut. Therefore, further research regarding the effect of PQQ on jejunal microbial communities should be conducted.

In summary, based on jejunal morphology, mucosal barrier function, immune response, colonic microflora, and SCFA levels, we conclude that intestinal health in piglets was damaged following the ETEC K88 challenge. Dietary supplementation with PQQ can effectively promote recovery of the jejunal mucosa following inflammatory injury by inhibiting the NF-κB pathway and correcting colonic microbiota dysbiosis in piglets challenged with ETEC K88.

## Data Availability Statement

The raw reads were deposited into the NCBI Sequence Read Archive (SRA) database (Accession Number: SRP218537).

## Ethics Statement

The animal study was reviewed and approved by all experimental protocols of the Animal Care and Use Committee of China Agricultural University (AW03059102-1) and carried out in compliance with the National Research Council’s Guide for the Care and Use of Laboratory Animals.

## Author Contributions

FW contributed to securing financial support, designing the study, and preparing the manuscript draft. CH contributed to analyzing samples, performing statistical analysis, and preparing the first manuscript draft. XM and YH contributed to designing the study and preparing the manuscript draft. DM, WW, and ZW carried out the animal feeding trial and sample collection. All authors contributed to the article and approved the submitted version.

## Conflict of Interest

The authors declare that the research was conducted in the absence of any commercial or financial relationships that could be construed as a potential conflict of interest.

## Abbreviations

ALP: alkaline phosphatase
CD: crypt depth
DAO: diamine oxidase
ETEC: enterotoxigenic *Escherichia coli*
GSH-Px: glutathione peroxidase
I κ B: inhibitor of nuclear factor-kappa B alpha
MDA: malondialdehyde
MyD88: myeloid differentiation primary response gene 88
NF- κ B: nuclear factor-kappa B
PQQ: pyrroloquinoline quinone
SIgA: secretory immunoglobulin A
T-AOC: total antioxidant capability
TLR4: toll like receptor 4
T-SOD: total superoxide dismutase
VCR: villus height/crypt depth
VH: villus height
ZO-3: zonula occludens-3.
