# Supplementary material for: Pyrroloquinoline Quinone Alleviates Jejunal Mucosal Barrier Function Damage and Regulates Colonic Microbiota in Piglets Challenged With Enterotoxigenic Escherichia coli
Source: Front Microbiol. 2020 Jul 24;11:1754. doi: 10.3389/fmicb.2020.01754 (PMC7396494; doi:10.3389/fmicb.2020.01754)
Supplement: Supplementary file 1 [file Table_1.DOCX]

**Supporting information Table S1 Ingredients and nutrition levels of diets (as-fed basis)**

| **Ingredients (%)** | **CTRL** | **PQQ** | **Nutrient levels** |  |
| --- | --- | --- | --- | --- |
| Corn | 59.34 | 59.04 | ME (Mcal/kg) | 3.61 |
| Soybean meal | 15.00 | 15.00 | Crude protein (%) | 18.50 |
| Extruded full-fat soybean | 5.00 | 5.00 | Calcium (%) | 0.79 |
| Soy protein concentrate | 2.00 | 2.00 | Digestible phosphorus (%) | 0.40 |
| Fish meal | 3.00 | 3.00 | Lys (%) | 1.35 |
| High protein whey powder | 6.00 | 6.00 | Met + cys (%) | 0.69 |
| Spray-dried plasma | 2.00 | 2.00 | Thr (%) | 0.84 |
| Sucrose | 2.00 | 2.00 | Try (%) | 0.25 |
| Soybean oil | 2.20 | 2.20 |  |  |
| Limestone | 0.90 | 0.90 |  |  |
| Dicalcium phosphate | 1.00 | 1.00 |  |  |
| Salt | 0.25 | 0.25 |  |  |
| Compound Amino Acid | 0.81 | 0.81 |  |  |
| PQQ pyrroloquinoline quinone | 0.00 | 0.30 |  |  |
| Premix(free-antibiotics) | 0.50 | 0.50 |  |  |

a) Premix provided per kilogram of the complete diet: vitamin A, 10000 IU; vitamin D3, 1500 IU; vitamin E, 30 IU; vitamin K3, 2.5 mg; vitamin B1, 1.5 mg; vitamin B2, 10 mg; vitamin B6, 10 mg; vitamin B12, 0.05 mg; folic acid, 1 mg; biotin,0.5 mg; niacin, 30 mg; pantothenic acid, 20 mg; Cu, 20 mg; Fe, 100 mg; Zn, 110 mg; Mn, 40 mg; Se, 0.3 mg; and I, 0.54 mg

**Supporting information Table S2 Effect of PQQ on growth performance in pigs**

| Items | CTRL | PQQ | SEM | P value |
| --- | --- | --- | --- | --- |
| Initial body weight, kg | 7.91 | 7.91 | 0.192 | 0.96 |
| Final body weight, kg | 12.45 | 12.61 | 0.51 | 0.56 |
| Average daily feed intake, g | 520 | 528 | 19 | 0.80 |
| Average daily gain, g | 324 | 344 | 11 | 0.29 |
| Average daily feed intake / Average daily gain | 1.61 | 1.53 | 0.02 | 0.01 |

a) CTRL for piglets fed the basal diet, PQQ for piglets fed the basal diet supplemented with PQQ, n=5

**Supporting information Table S3 Primer sequences of target and reference gene**

|  | **Accession number** | **Primer sequences (5' to 3')** | **Product size (bp)** |
| --- | --- | --- | --- |
| β-actin | XM_021086047.1 | F CCACGAAACTACCTTCAACTC | 131 |
|  |  | R TGATCTCCTTCTGCATCCTGT |  |
| TLR4 | NM_001113039.2 | F CTCCAGCTTTCCAGAACTGC | 192 |
|  |  | R AGGTTTGTCTCAACGGCAAC |  |
| MyD88 | NM_001099923.1 | F ATTGAAAAGAGGTGCCGTCG | 188 |
|  |  | R CAGACAGTGATGAACCGCAG |  |
| NF-Kb | NM_001048232.1 | F CTCGCACAAGGAGACATGAA | 147 |
|  |  | R ACTCAGCCGGAAGGCATTAT |  |
| IL-1β | XM_021085847.1 | F TTGTCTGTGATGCCAACGTG | 108 |
|  |  | R TGAGGAGGTGGAGAGCCTTC |  |
| TNF-α | NM_214022.1 | F CCAATGGCAGAGTGGGTATG | 116 |
|  |  | R TGAAGAGGACCTGGGAGTAG |  |
| IL-17 | NM_001005729.1 | F CAGCAAGCTCCAGCTCATCCATC | 92 |
|  |  | R CAGCAGAAGCAGCAGTGACAGG |  |
| IL-4 | NM_214123.1 | F GCTTCGGCACATCTACAGACACC | 110 |
|  |  | R TCTTGGCTTCATGCACAGAACAGG |  |
| IL-10 | NM_214041.1 | F AACCACAAGTCCGACTCAACGAAG | 81 |
|  |  | R GCCAGGAAGATCAGGCAATAGAGC |  |
| IL-6 | NM_001252429.1 | F ACCGGTCTTGTGGAGTTTCA | 170 |
|  |  | R GCATTTGTGGTGGGGTTAGG |  |
| INF-γ | NM_213948.1 | F AGCTTTGCGTGACTTTGTGT | 152 |
|  |  | R ATGCTCCTTTGAATGGCCTG |  |

a) F, forward primer; R, reverse primer.

**
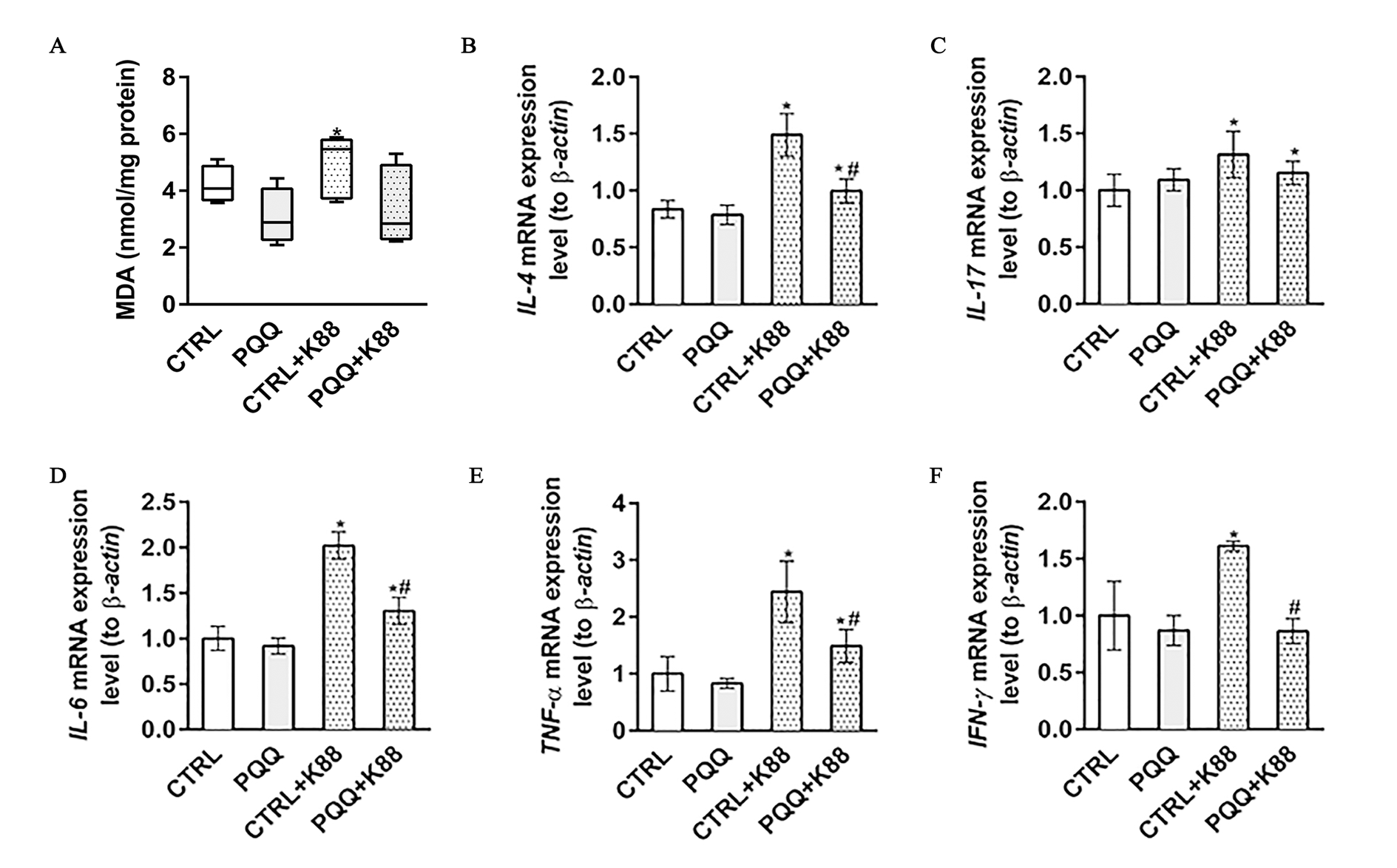
**

**
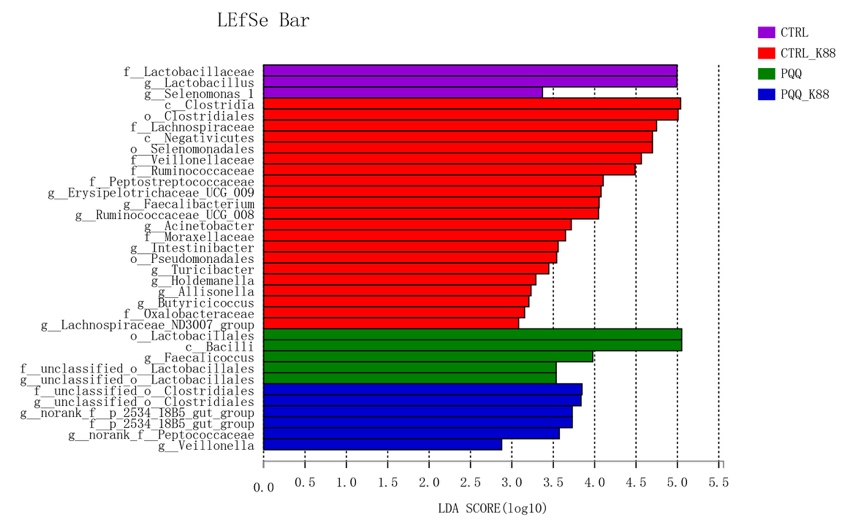

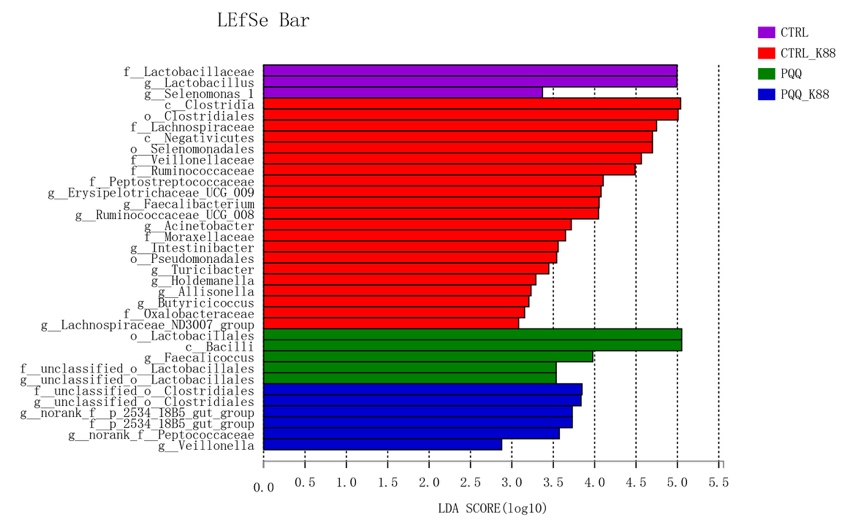

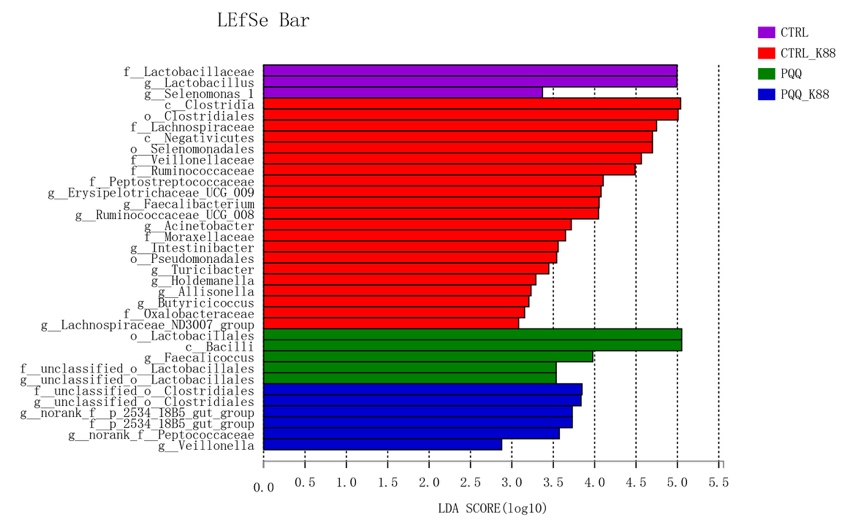

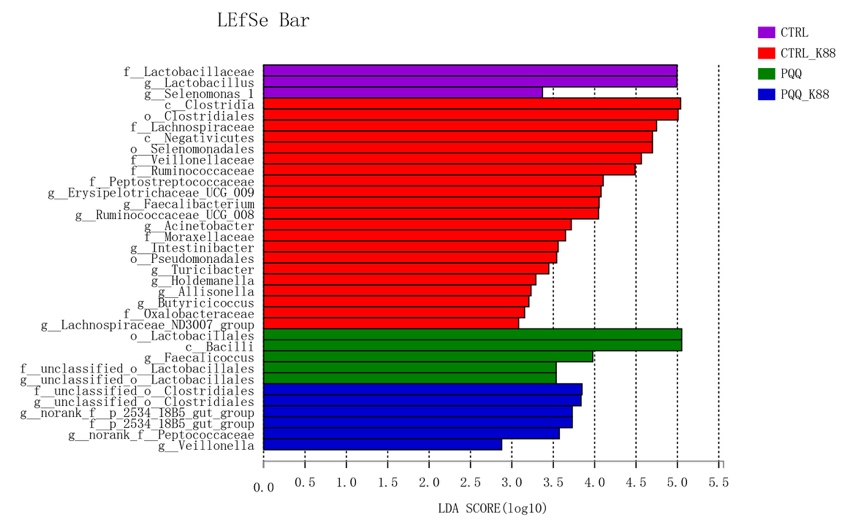

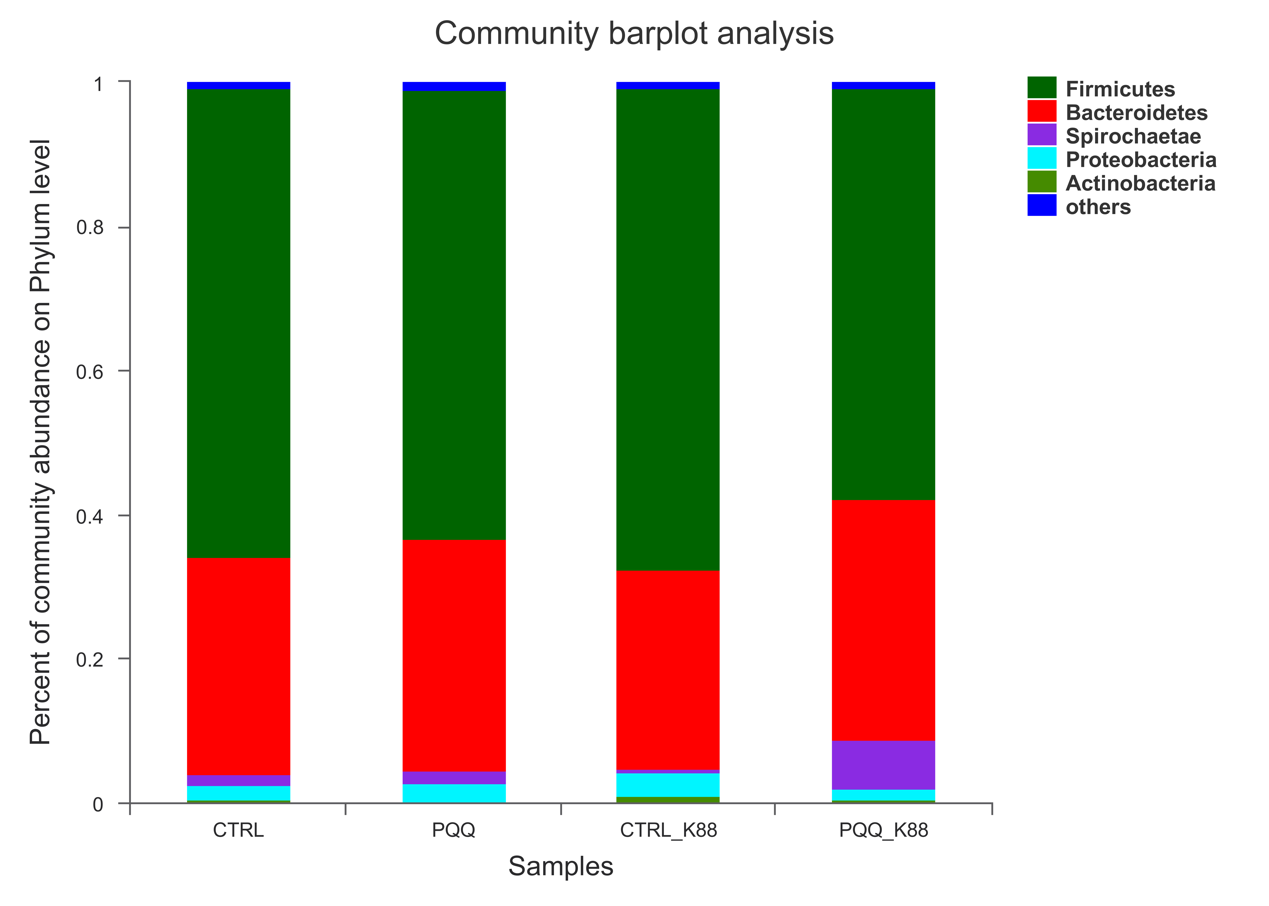

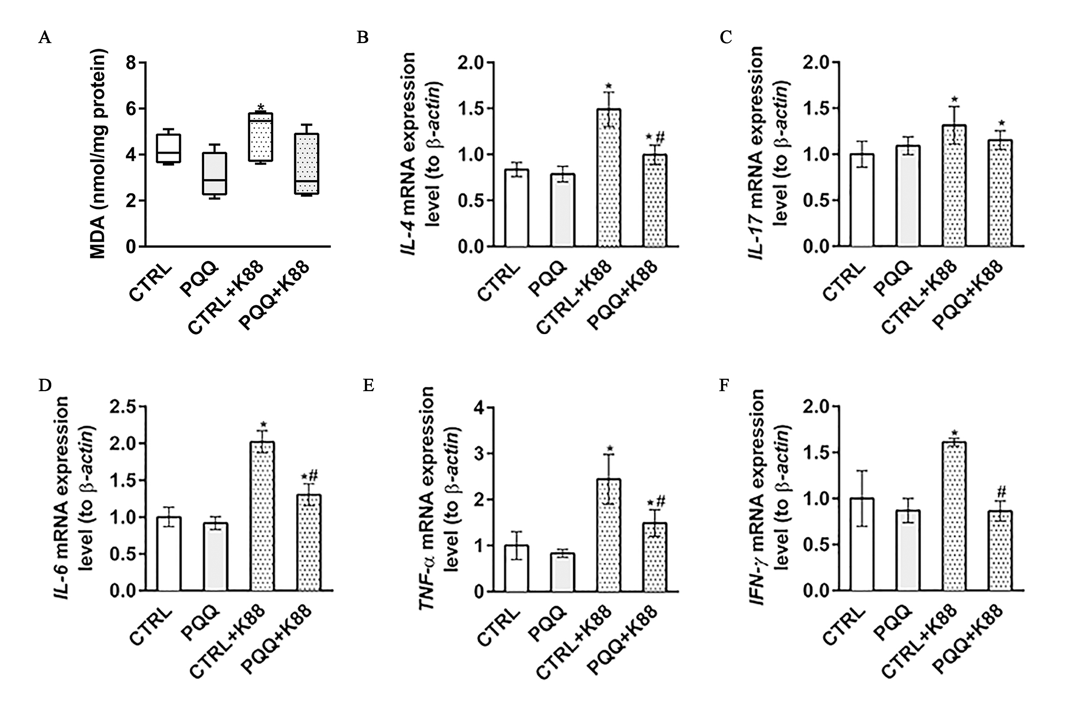

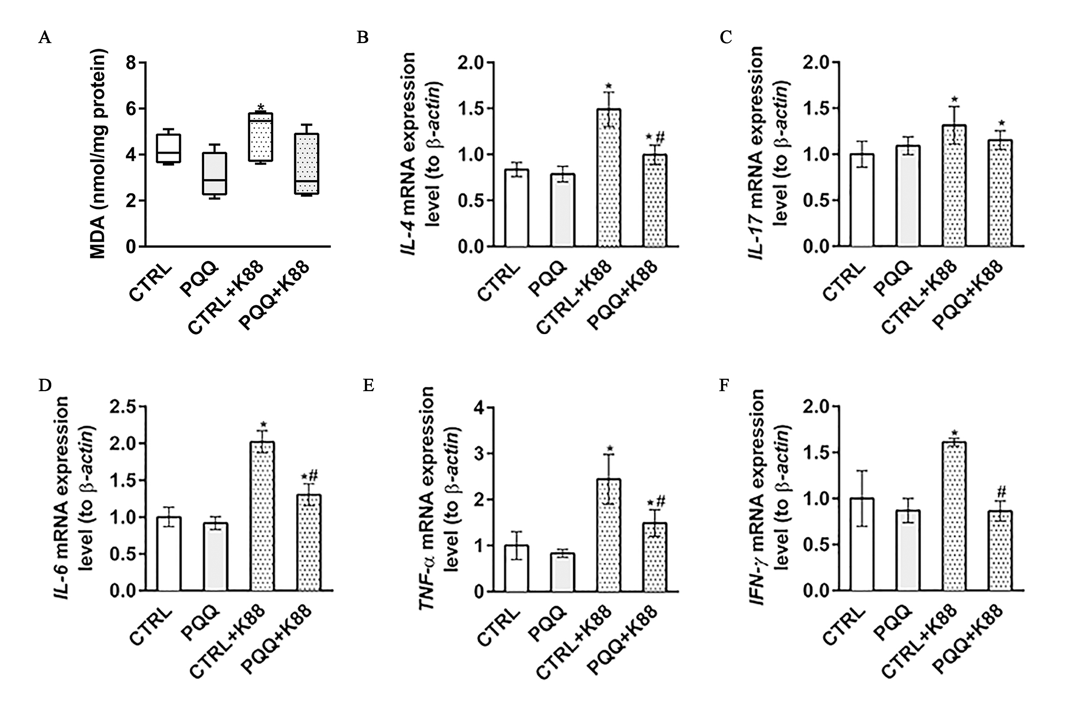

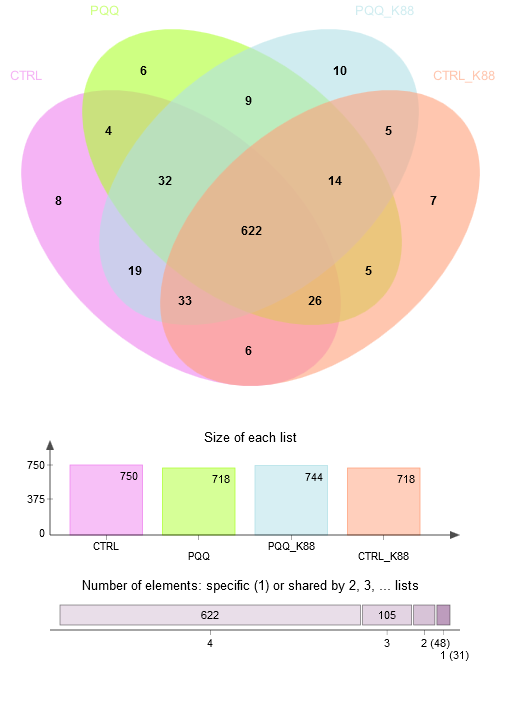

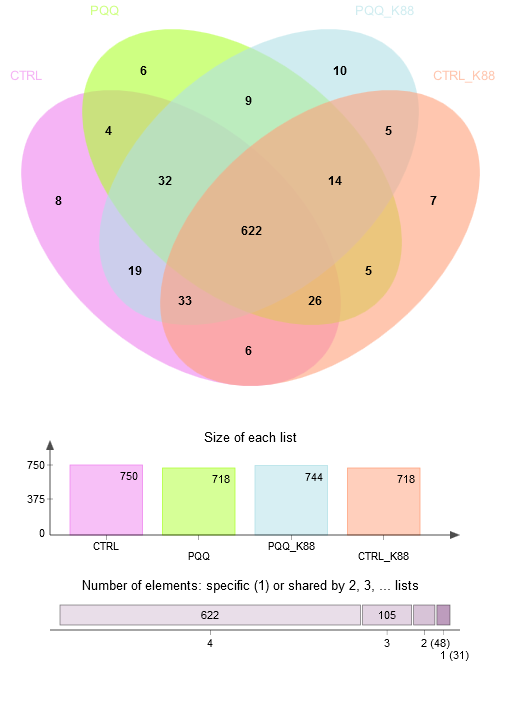

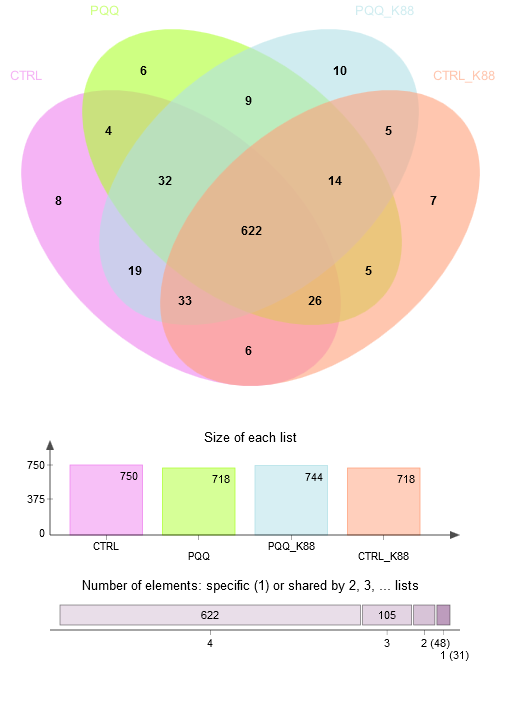

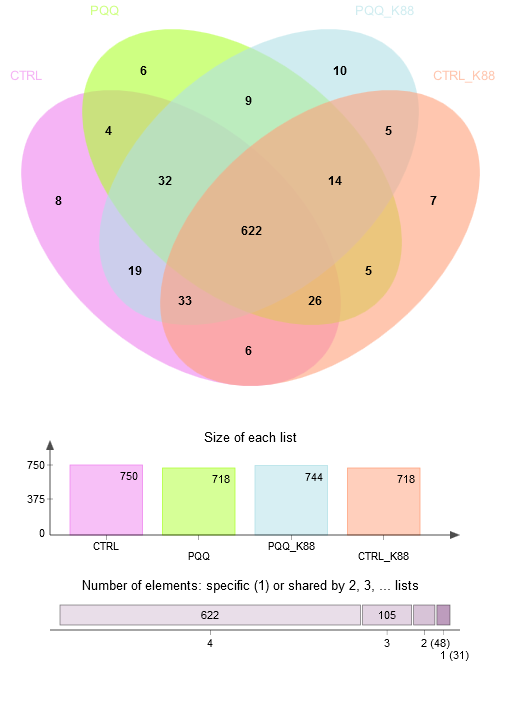

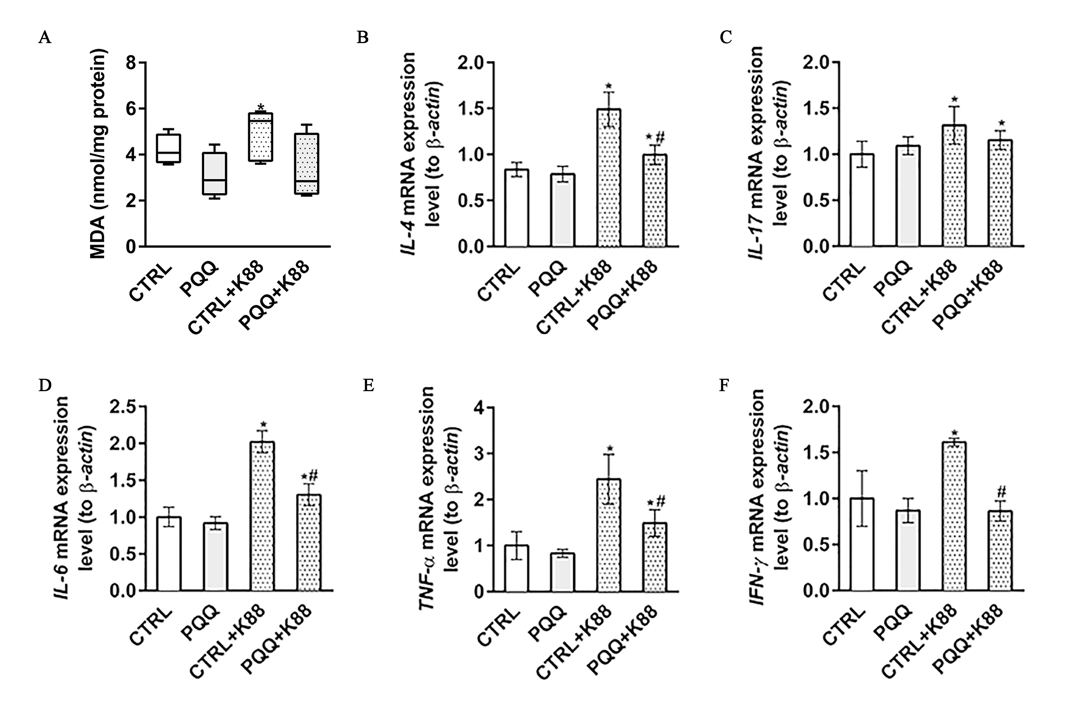

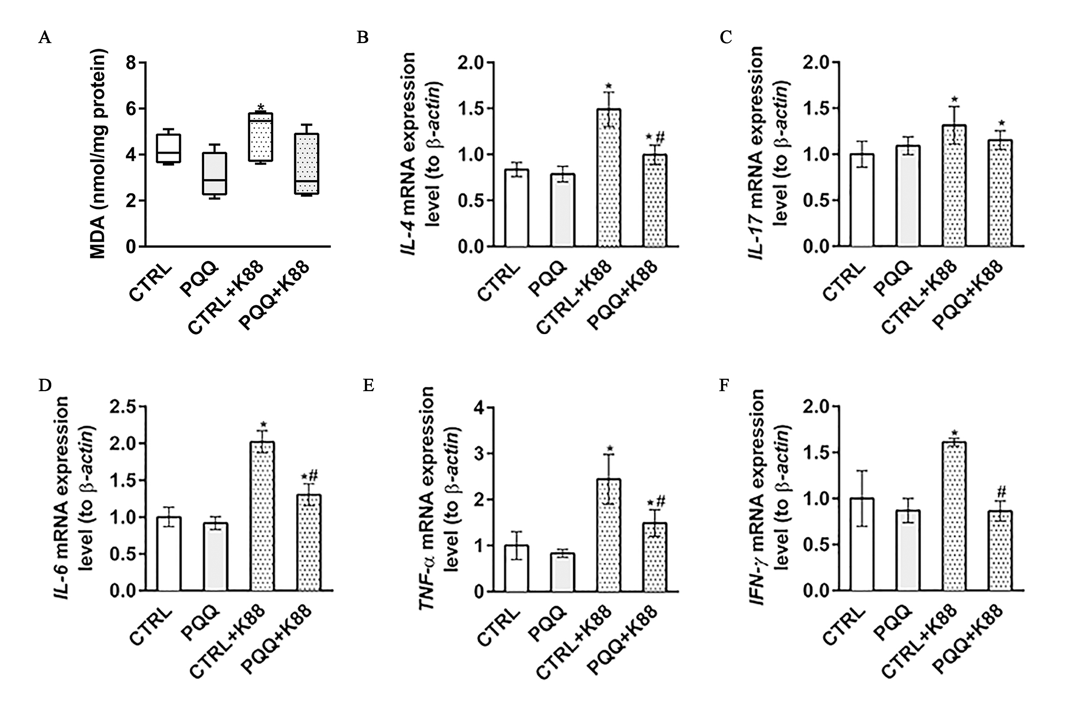

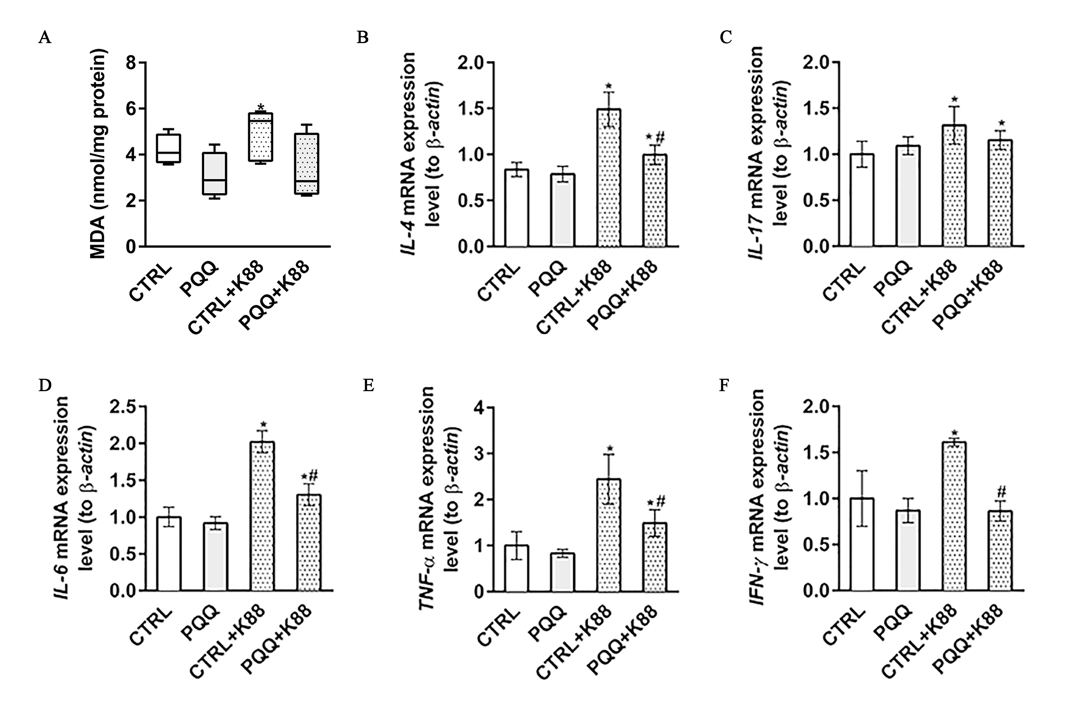

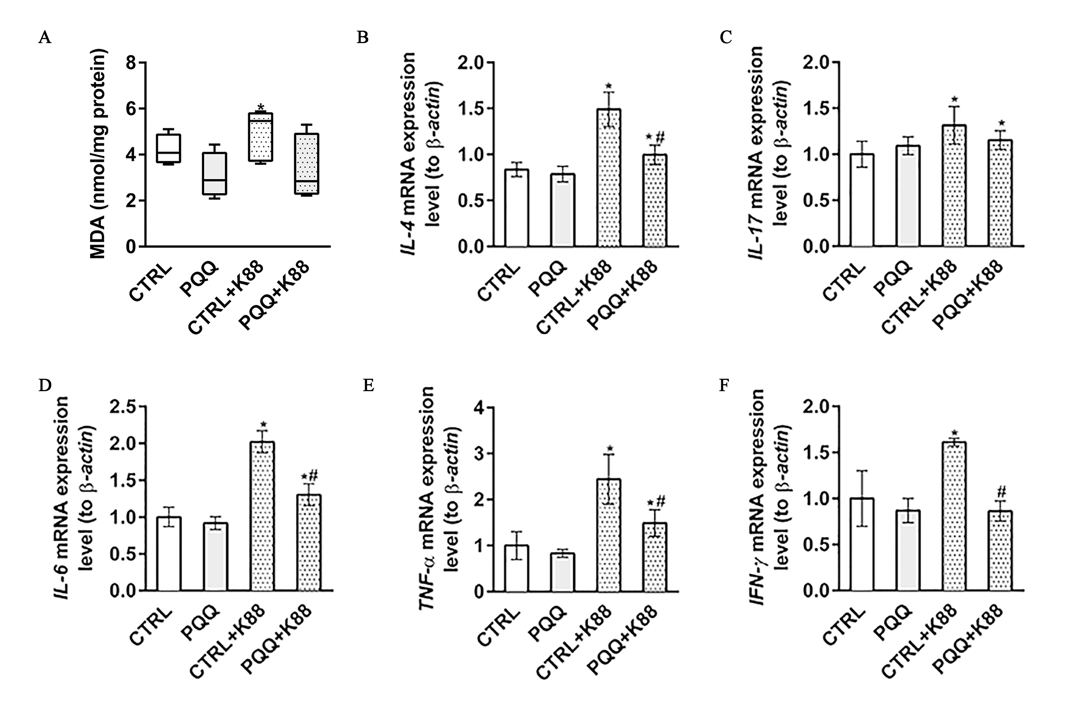

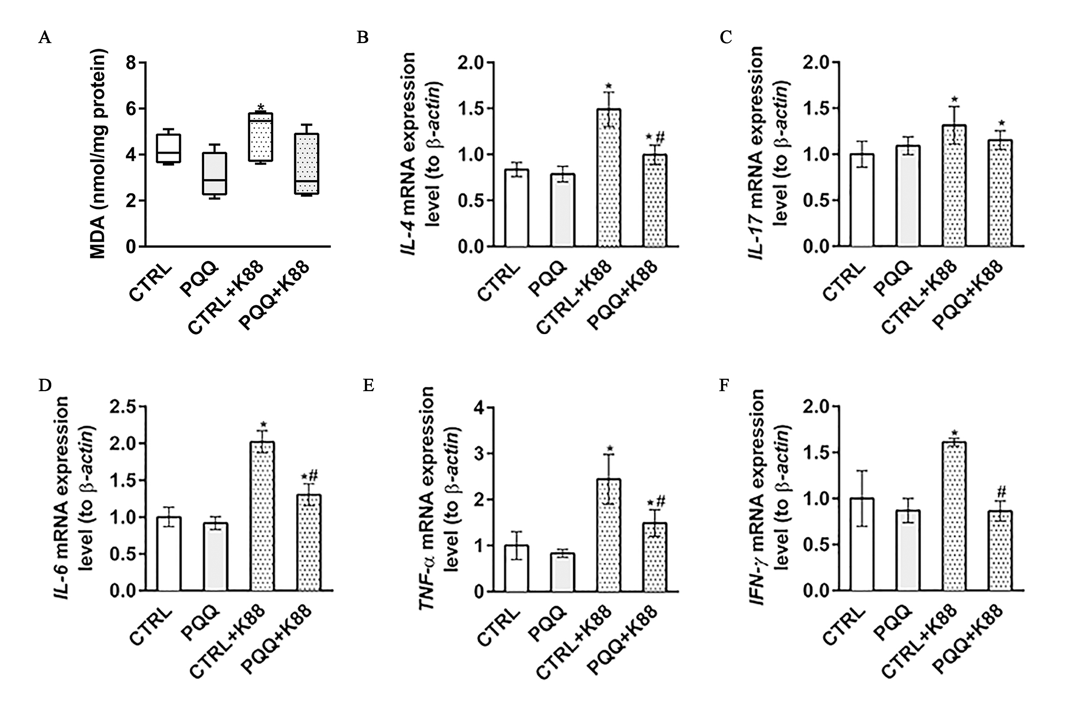

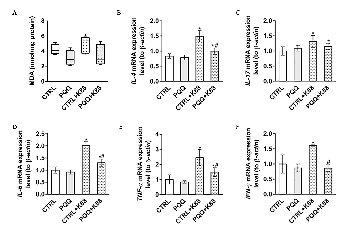

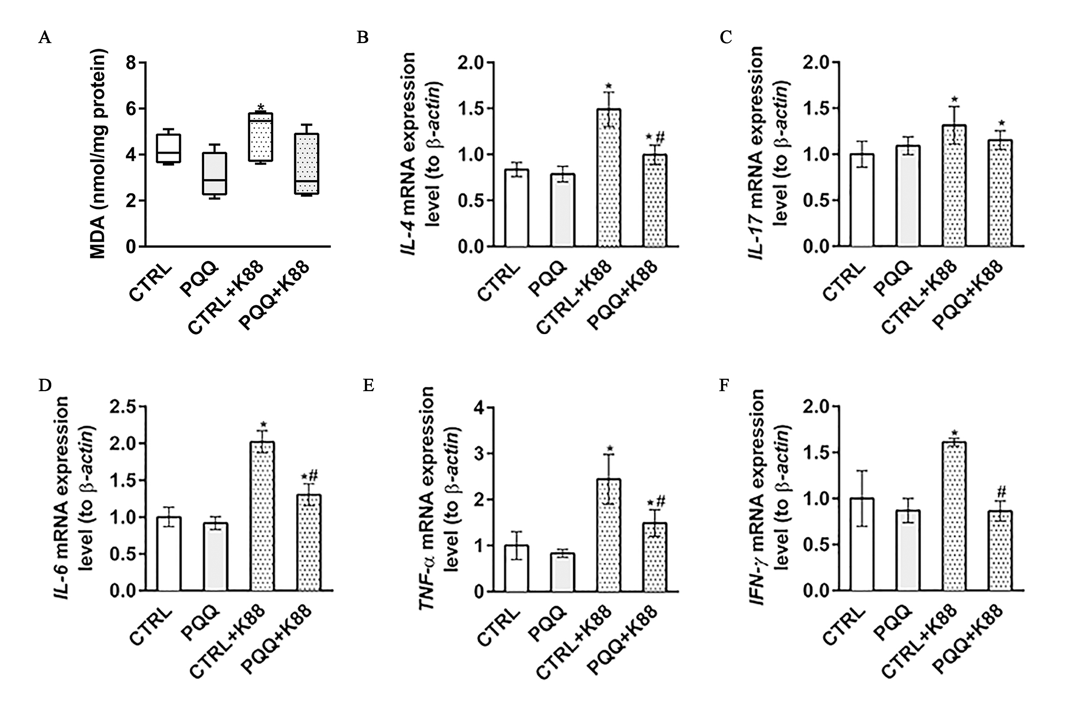

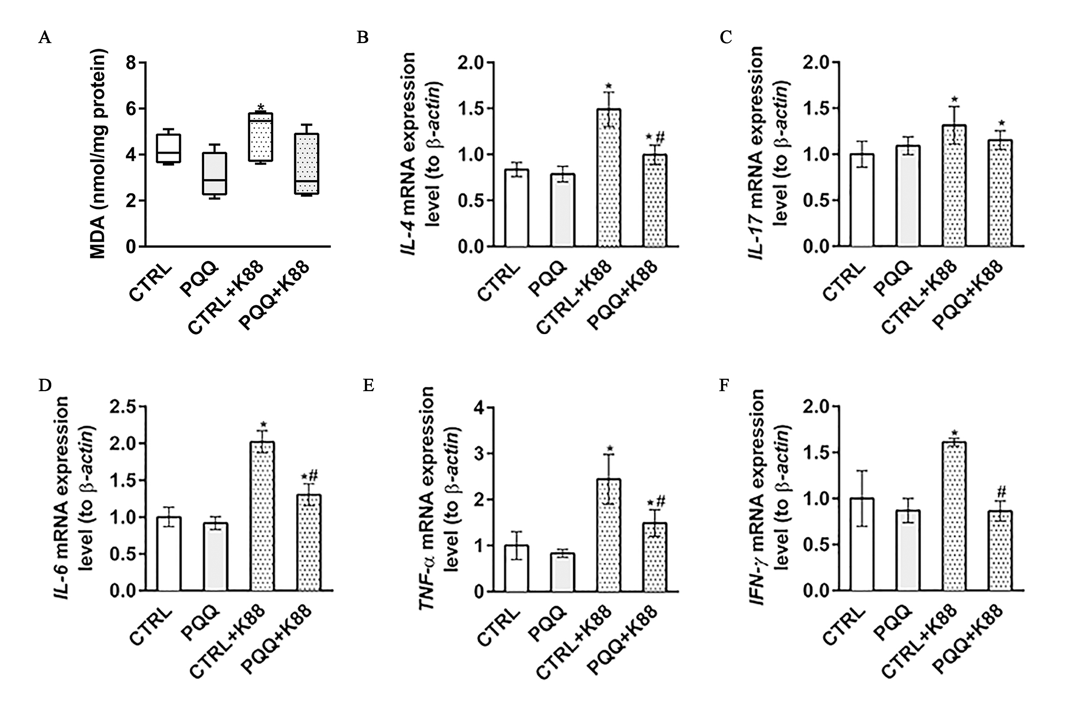

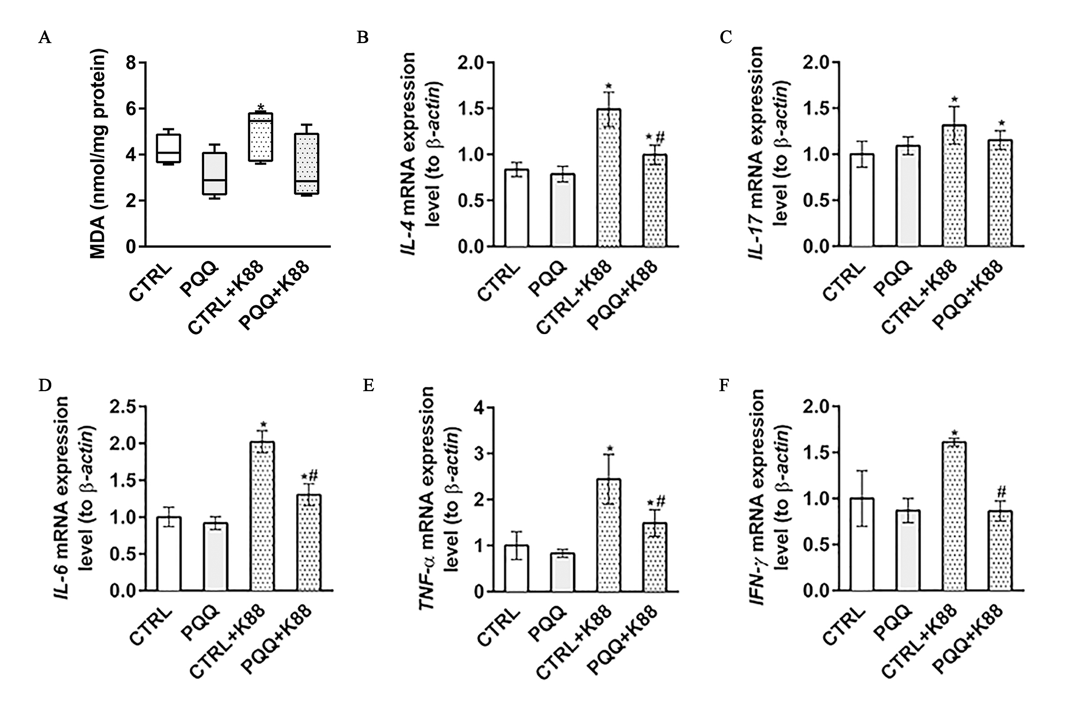

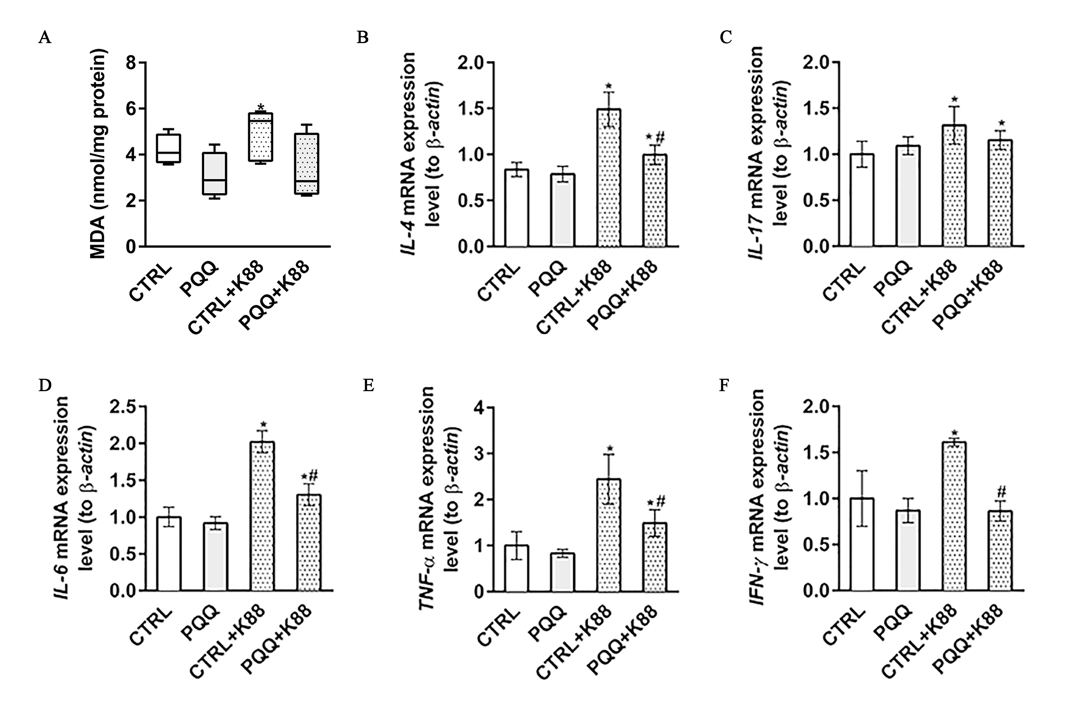
**

S**upporting information Figure S1 Effects of PQQ supplementation on mucosa cytokines expression in jejunum after ETEC K88 challenge in weaned pigs.** (A) Elisa for MDA level, (B-F) real-time PCR for inflammatory factors. β-actin was the housekeeping gene. piglets were untreated or infected with ETEC, or fed with PQQ either alone or together with ETEC for 24 h. CTRL for piglets fed the basal diet, PQQ for piglets fed the basal diet supplemented with PQQ, CTRL + K88 for piglets fed basal diet and challenged with ETEC K88, PQQ + K88 for the piglets fed basal diet supplemented with PQQ and challenged without ETEC K88.* denote significant (P < 0.05) difference from the CTRL group. # denote significant (P < 0.05) difference from the CTRL+ K88 group, n=5.

**B**

**A**


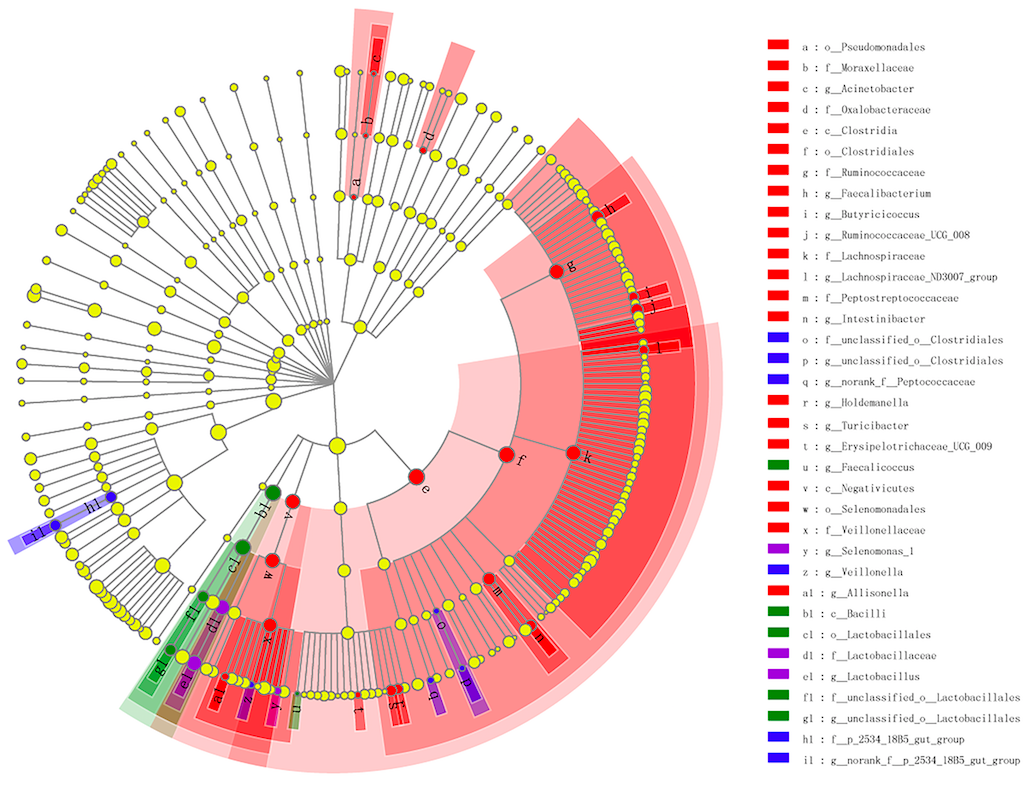
**
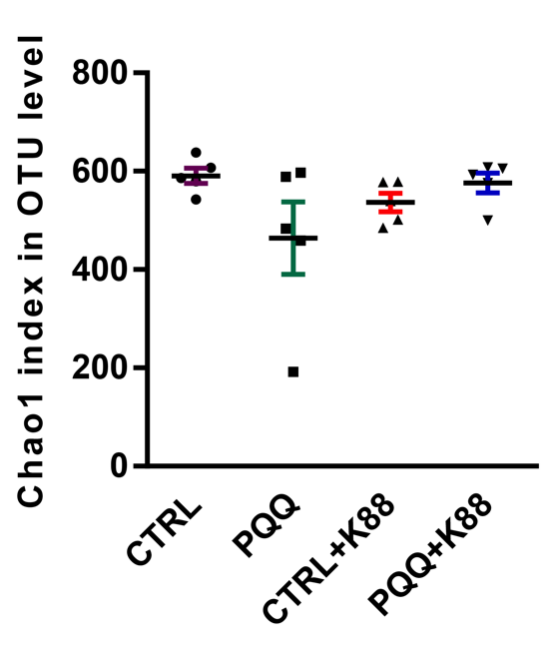

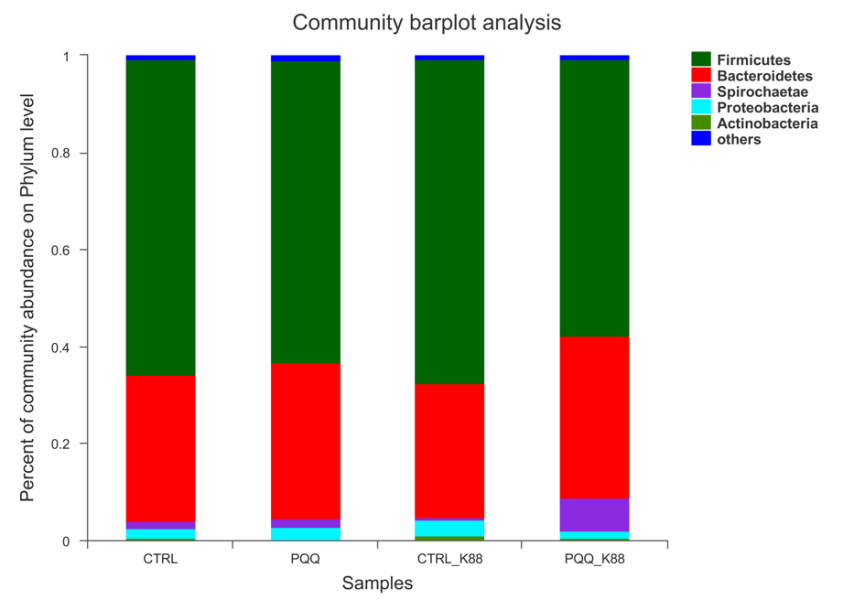

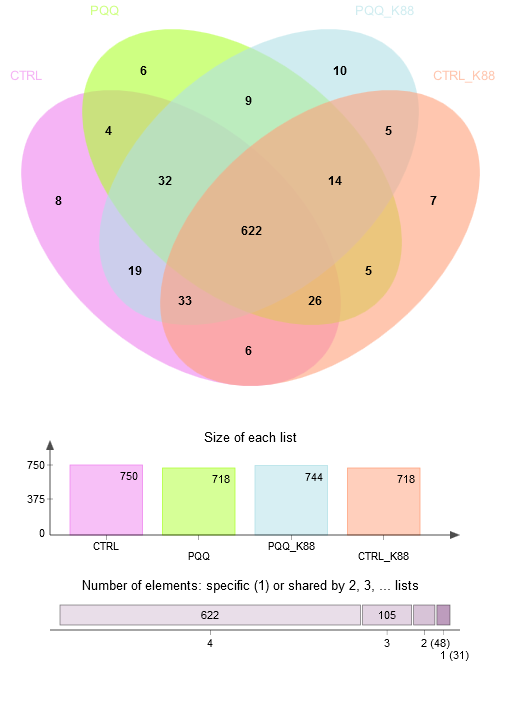
** **Supporting information Figure S2 Composition of the colonic microbiota after PQQ supplementation in ETEC K88 challenged piglets**. (A) The number of OTUs in the four groups were represented by venn diagram. (B) The α diversity for chao 1 index showed the diversity and richness of colonic microbiota in CTRL, PQQ, CTRL+K88 and PQQ + K88 groups. (C) Relative contribution of phyla in the CTRL, PQQ, CTRL+K88 and PQQ + K88 groups in the colon. (D) LEfSe sequence analysis reflected taxonomic cladogram in the colonic. (H) LEfSe sequence analysis reflects taxonomic cladogram of colonic microbiota. Colored circles and shaded parts represent biomarker taxa. Taxa in the community are shown by the diameter of each circle.

**C**

**D**
